# Genetic differentiation of European grayling (*Thymallus thymallus*) populations in Serbia, based on mitochondrial and nuclear DNA analyses

**DOI:** 10.1186/1297-9686-43-2

**Published:** 2011-01-14

**Authors:** Saša Marić, Andrej Razpet, Vera Nikolić, Predrag Simonović

**Affiliations:** 1 University of Belgrade, Faculty of Biology, Institute of Zoology, Studentski trg 16, 11001 Belgrade, Serbia; 2 University of Ljubljana, Biotechnical Faculty, Department of Animal Science, Groblje 3, 1230 Domžale, Slovenia

## Abstract

**Background:**

The structure and diversity of grayling (*Thymallus thymallus*) populations have been well studied in most of its native habitat; however the southernmost populations of the Balkan Peninsula remain largely unexplored. The purpose of this study was to assess the genetic diversity of Serbian grayling populations, detect the impact of stocking and provide guidelines for conservation and management.

**Methods:**

Eighty grayling individuals were collected from four rivers (Ibar, Lim, Drina and Rzav). The mitochondrial DNA control region (CR; 595 bp of the 3'end and 74 bp of flanking tRNA) and the ATP6 gene (630 bp fragment) were sequenced for 20 individuals (five from each locality). In addition, all individuals were genotyped with 12 microsatellite loci. The diversity and structure of the populations as well as the recent and ancient population declines were studied using specialized software.

**Results:**

We detected three new haplotypes in the mtDNA CR and four haplotypes in the ATP6 gene of which three had not been described before. Previously, one CR haplotype and two ATP6 gene haplotypes had been identified as allochthonous, originating from Slovenia. Reconstruction of phylogenetic relations placed the remaining two CR haplotypes from the River Danube drainage of Serbia into a new clade, which is related to the previously described sister Slovenian clade. These two clades form a new Balkan clade. Microsatellite marker analysis showed that all four populations are genetically distinct from each other without any sign of intra-population structure, although stocking of the most diverse population (Drina River) was confirmed by mtDNA analysis. Recent and historical population declines of Serbian grayling do not differ from those of other European populations.

**Conclusions:**

Our study shows that (1) the Ibar, Lim and Drina Rivers grayling populations are genetically distinct from populations outside of Serbia and thus should be managed as native populations in spite of some introgression in the Drina River population and (2) the Rzav River population is not appropriate for further stocking activities since it originates from stocked Slovenian grayling. However, the Rzav River population does not represent an immediate danger to other populations because it is physically isolated from these.

## Background

The recent natural dispersal area of the European grayling (*Thymallus thymallus*) extends westward to France and Great Britain, northward across Fenoscandinavia and northern Russia, eastward to the Ural Mountains near the Kara River [1] and southward to the headwaters in the drainage areas of Ibar (Serbia) and Lim (the Ljuča River, Montenegro) Rivers in the western Balkans.

Fossil evidence suggests that European grayling inhabited Europe long before the Pleistocene cold periods [2], corroborating the pre-glacial introgression of grayling and its expansion across Europe, as also suggested by Weiss et al. [3]. Numerous DNA marker-based studies on population genetic structure, phylogeography and phylogeny of European grayling are now available for various geographic regions (e.g. [3-8]), as well as on local scales (e.g. [9-18]). Studies on the matrilineal phylogeography and post-glacial dispersal routes of European grayling have revealed 27 haplotypes in the ND-5/6 and cyt-b/D-loop (CR) regions of mitochondrial DNA [5], 58 haplotypes in the D-loop (CR) region [3] and 30 ND-1 haplotypes in the ND-5/6 gene region [7]. All the results suggest the existence of distinct Danubian clades, as well as Central-Eastern, Central-Western, Northern/Northeastern and mixed clades [19]. Rather distinct grayling clades were detected in the Adriatic region and in the Loire basin with a single haplotype (At1) that is highly divergent compared to those of the remaining clades [3]. The assumed refugial region for (i) the Northern/Northeastern-European clade was the area north of the Caspian and Black Seas, (ii) the Central-Eastern European clade, the ice-free tributaries of Vistula and Elbe Rivers, (iii) the Central-Western Europe, the ice-free tributaries of Rhine, Main and upper Danube, and (iv) the Danubian clades, the lower Danube drainage area, i.e., in the Balkan Peninsula [19]. Based on CR mtDNA sequence analyses and calibration of molecular clock applied to the nucleotide divergence of these sequences between the major grayling clades with a CR mutation rate of 1% per million years (MY), Froufe et al. [4] have dated the colonization of Europe to the Pliocene-Pleistocene boundary around 4.6 to 1.6 million years ago (MYA), far before the onset of Pleistocene ice age. However, during the late Pleistocene and Holocene glaciations, it is assumed that secondary contacts occurred in all drainages, e.g. in the upper reaches of the rivers Main, Danube, Elbe and Rhine (Lake Constance) [19]. Koskinen et al. [6] have revealed that a substantial proportion of molecular variation (44%) in European grayling exits between populations, whereas Gum et al. [7,16] have revealed that about 25% of the total genetic variation is explained by differences between major drainage systems, about 11 to 20% by differences between populations within drainages and about 57 to 64% by differences within populations.

The Balkan Peninsula along with the Apennine and Iberian Peninsulas, were a refuge area during the Pleistocene glaciations and therefore might represent crossroads of different evolutionary patterns and processes [20,21]. The Balkan Peninsula, in contrast to the Apennine and Iberian Peninsulas, is poorly explored (except Slovenia). This part of Europe, very important for post-glacial faunal evolution and colonization, is noted as a biodiversity hotspot [22,23]. The last (Würm) glaciation in Europe ended ~10 000 years ago coinciding with both colonization of the present grayling habitat and decline of grayling populations. Based on the 34 European populations (none from the Balkans), between 1000 to 10000 years ago, population sizes were reduced to 0.03-1.2% of their historical sizes [24].

An even more recent decline of European grayling populations throughout central Europe, due to pollution, habitat destruction, river engineering, predation from piscivorous birds and overfishing [1,13,15,25-27], is also characteristic to Serbian grayling populations. Janković [28] has reported results for six Serbian rivers with grayling populations (Drina, Lim, Uvac, Jadar, Studenica and Ibar). The populations from Uvac, Jadar and Studenica Rivers went extinct, while one new population was established in the Rzav River through stocking with fish originating from an unknown source population from Slovenia. Population decline leads to an increase in management activities that involve rearing and stocking grayling, which may cause a change in genetic architecture and extinction of natural populations [29].

The main goal of the present study was to investigate the genetic diversity of grayling populations in Serbia, using two mtDNA loci (CR and ATP6), in order to clarify the phylogeography of grayling populations in this previously unstudied part of its native range. Additionally, 12 microsatellite loci were analyzed, in order to (i) characterize the genetic variability and differentiation, (ii) compare recent and historical declines in previously studied European populations [24] with that of Serbian populations and (iii) examine whether it is possible to identify non-introgressed indigenous populations of grayling for future management and supportive breeding.

## Methods

### Sampling and DNA isolation

**Figure 1:**
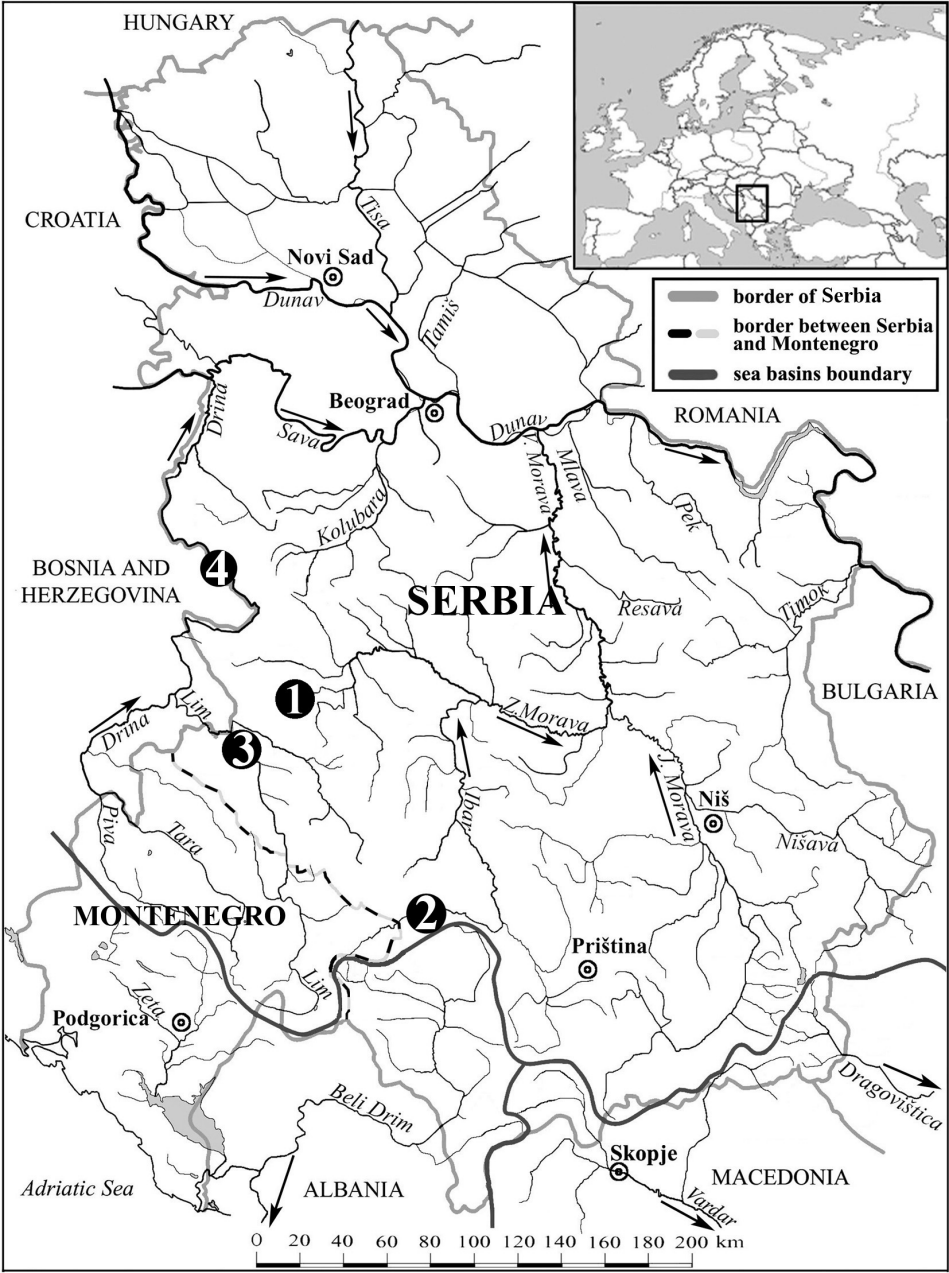
**Sampling locations in Serbia**. Names of sampling sites are listed in Table 1

Eighty grayling individuals from four Serbian locations across the Danubian drainage were collected by electrofishing and angling between 2007 and 2008 (Table 1 and Figure 1). Fin clips were sampled and stored in 96% ethanol. Total DNA was isolated from this tissue using the Wizard Genomic DNA Purification Kit (Promega), following the supplier's instructions.

**Table 1:** Sample locations with a summary of mtDNA haplotype frequencies (all underlined haplotypes are described for the first time)

Haplotype frequency													
	**Nb samples**		**ATP6 gene**				**Control Region**			**Combined haplotypes**			

**Code populations**	**msDNA**	**mtDNA**	**Slo**	**Soc18**	**Bal**	**BoDr**	**Da25**	**Da27**	**Da29**	**Da25Slo**	**Da25Soc18**	**Da27Bal**	**Da29BoDr**

**1. Rzav**	20	5	1	4	-	-	5	-	-	1	4	-	-
**2. Lim**	20	5	-	-	5	-	-	5	-	-	-	5	-
**3. Ibar**	20	5	-	-	5	-	-	5	-	-	-	5	-
**4. Drina**	20	5	2	-	2	1	2	2	1	2	-	2	1

∑	**80**	**20**	**3**	**4**	**12**	**1**	**7**	**12**	**1**	**3**	**4**	**12**	**1**

Sequences of the newly described haplotypes are available in GenBank under Accession numbers: Bal: HM641130, BoDr: HM636920, Slo: HM636921, Da25: HM636922, Da27: HM636923 and Da29: HM636924

Fifty-seven haplotypes from GenBank (accession numbers AF522395-AF522452) were used for the phylogenetic analysis and outgroup taxa included three individuals of *Thymallus arcticus *(AF522453), *Thymallus grubei *(AF522454) and *Thymallus brevirostris *(AF522455).

The number of geographical rivers sampled for Serbian grayling in this study was limited and could not be increased because the four rivers included are the only ones remaining in Serbia today, with native grayling occurring only in three of these (see Introduction, Janković [28]).

### Mitochondrial DNA sequence analysis

Two mtDNA loci, control region (CR) and ATP6 were amplified in 20 individuals by polymerase chain reaction (PCR). The complete CR [1043 base pairs (bp)] and 162 bp of the flanking tRNA were amplified using the LRBT-25 and LRBT-1195 primers [15]. The complete ATP6 gene (648 bp) was amplified using the L8558 and H9208 primers [30].

The following PCR conditions were used: each reaction mixture (30 μL) contained 21.6 μL H_2_O (21 μL H_2_O for ATP6), 3 μL 10 × PCR buffer, 0.75 μL 10 mM of each primer, 1.2 μL 25 mM MgCl_2 _(1.8 μL 25 mM MgCl_2 _for ATP6), 1.5 μL 0.2 mM dNTP, 0.2 μL Fermentas *Taq *polymerase and 1 μL of template DNA (~100 ng of genomic DNA); the cycle parameters were as follows: initial denaturation (95°C, 3 min) followed by 32 cycles of strand denaturation (95°C, 45 s), primer annealing (55°C, 45 s) and DNA extension (72°C, 60 s). All PCR amplifications were performed in a programmable thermocycler GeneAmp^® ^PCR System 9700 (Applied Biosystems). Amplified DNA fragments were run on a 1.5% agarose gel and subsequently isolated from the gel using the QIAEX II gel Extraction Kit (QIAGEN).

All sequencing reactions were prepared using a BigDye Terminator v3.1 Cycle Sequencing Kit (Applied Biosystems) according to the manufacturer's recommendations. The 3'end of the 595 bp fragment of the mtDNA CR with the 74 bp of flanking tRNA were sequenced using primer LRBT-1195 [15]. The 5'end of the 630 bp ATP6 fragment was sequenced using primer L8558 [30]. The amplified, fluorescently labeled and terminated DNA was salt-precipitated and analyzed on an ABI Prism 3130xl Genetic Analyzer.

### Microsatellite marker analysis

Twelve microsatellite loci were isolated and characterized as previously described i.e. BFRO004 [31], BFRO005 to BFRO008 [9], BFRO010 and BFRO011 [32], BFRO013 [11], BFRO015 to BFRO018 [33]. They were amplified in 80 individuals using fluorescently labeled forward primers. The following PCR conditions (10 μL reactions) were used: 6.325 μL H_2_O, 1 μL 10 × PCR buffer, 0.25 μL 10 mM of each primer, 0.6 μL 25 mM MgCl_2_, 0.5 μL 0.2 mM dNTP, 0.075 μL Fermentas *Taq *polymerase and 1 μL of template DNA (~100 ng of genomic DNA); the cycle parameters were as follows: initial denaturation (94°C, 3 min) followed by 30 cycles of strand denaturation (94°C, 45 s), primer annealing (55°C, 30 s for BFRO004 to BFRO010, and 60°C, 30 s for BFRO011 to BFRO018) and DNA extension (72°C, 5 s). Fragment analysis was performed on a 3130xl Genetic Analyzer and genotyped using GeneMapper v4.0.

### Mitochondrial DNA data analysis

DNA sequences were aligned using the computer program ClustalW [34]. Sequence polymorphism was assessed using DNAsp ver. 4.10 [35] and sequence divergence was estimated by the net nucleotide divergence (D_a_) using MEGA version 2.1 [36].

Aligned haplotypes were imported into the program PAUP Version 4.0b10 [37] for phylogenetic analysis. Neighbour-Joining (NJ) and maximum parsimony (MP) analyses were carried out for phylogenetic reconstruction. For the NJ analysis, a HKY85 model of substitution was chosen. Support for the nodes was obtained with 1000 bootstrap replicates. For the MP analysis, insertions or deletions (indels) were included as a fifth character, and the inferred phylogeny was estimated with 50% majority rule consensus tree. A heuristic search (1000 replicates) with Tree Bisection Reconnection (TBR) branch-swapping was employed to find the most parsimonious trees.

Relations among haplotypes were also determined using the TCS 1.2 program [38] with a connection limit fixed at 24 steps to include all the different haplotypes. Gaps were analyzed as a fifth character.

### Microsatellite marker data analysis

Microsatellite allele frequencies, expected (H_E_), non-biased (H_n.b_) and observed (H_O_) heterozygosities were calculated with GENETIX 4.04 [39]. FSTAT 2.9.3.2 [40] was used to calculate deviations from Hardy-Weinberg expectations (HWE), allelic richness and pair-wise F_ST _values, all based on 1000 permutations. Genetic relationships between individuals were estimated as the proportion of shared alleles at each locus, i.e. allele sharing distances (D_AS_) [41]. A matrix of D_AS _was used to construct Neighbour-Joining trees of individuals and populations with POPULATIONS software [42].

Recent population declines (2N_e_-4N_e _generations ago) can be detected with BOTTLENECK 1.2.02 [43] using the recommended stepwise mutation model (SMM) and the two-phase model (TPM) with 95% of single-step mutations and variance mutation size set to 12. To detect historical population declines, the coalescent analysis approach implemented in MSVAR 1.3 assuming strict SMM was used [44]. For the exponential model, we followed the settings used by Swatdipong et al. [24] with a five-year generation time discarding the first 10% of 2 × 10^8 ^iterations.

Population structure was inferred using the STRUCTURE program [45], which seeks solutions for a given number of clusters K applied to genotypic data in such a way that each cluster is in or close to Hardy-Weinberg and linkage equilibrium [46]. For runs estimating ln Pr(X|K) under a certain K, different run lengths were used (from 20000 to 100000 burn-in and 100000-2000000 total length, repeated 7 times for each K) depending on convergence. We applied the ΔK method [47] to estimate the most probable K.

## Results

### Mitochondrial DNA sequence analysis

Three new haplotypes were detected by sequencing the mtDNA CR: Da25, Da27 and Da29. Haplotype Da25 was present in the Rzav River population with a 100% frequency and in the Drina River population with a 40% frequency. Haplotypes Da25 and Da24 share synapomorphies at positions 622, 625, 626 and 635, and at position 708 with haplotypes Da22 and Da23 (See additional file 1: Variable nucleotide positions for CR haplotypes). Haplotypes Da22, Da23 and Da24 were observed in the population of the Sava River drainage area in Slovenia [3]. Haplotype Da27 was dominant in the samples from all localities in Serbia, with a 100% frequency for the Ibar and Lim Rivers and a 40% frequency for the Drina River. Haplotype Da29 was present exclusively in the Drina River population with a 20% frequency. Haplotypes Da27 and Da29 differed at five polymorphic positions and their genetic distance is about 0.75%. Haplotype Da27 and Da29 and haplotype Da25 differed at nine and ten polymorphic positions, respectively, and the genetic distance between these is about 1.5%.

Sequencing of the ATP6 gene revealed four haplotypes (Soc18, Slo, BoDr and Bal). Haplotype Soc18 had already been described in the population of Soča River in Slovenia [4] while in Serbia it was found only in samples from the Rzav River with an 80% frequency. The other three haplotypes had never been described before. Haplotype Slo was present in the populations of the Rzav River with a 20% frequency and the Drina River with a 40% frequency. Haplotype Bal was dominant in the sample analyzed here and was present in the populations of the Ibar and Lim Rivers with a 100% frequency and in the Drina River with a 40% frequency. Haplotype BoDr was found only in the Drina River samples with a 20% frequency. Unlike the mtDNA CR for which up to 10 polymorphic positions were identified among the three haplotypes, only three polymorphic positions were detected in the four ATP6 haplotypes and all were silent mutations. The synapomorphic position 34 discriminated between haplotypes Soc18 and Slo and haplotypes Bal and BoDr, which differed by two polymorphic positions at most, i.e. by a genetic distance of up to 0.32% (See additional file 2: Variable nucleotide positions for ATP6 gene haplotypes). Combining three CR and four ATP6 haplotypes produced four combined haplotypes (Table 1), since the samples that possessed different ATP6 haplotypes (Soc18 and Slo) shared the same CR haplotype Da25.

**Figure 2:**
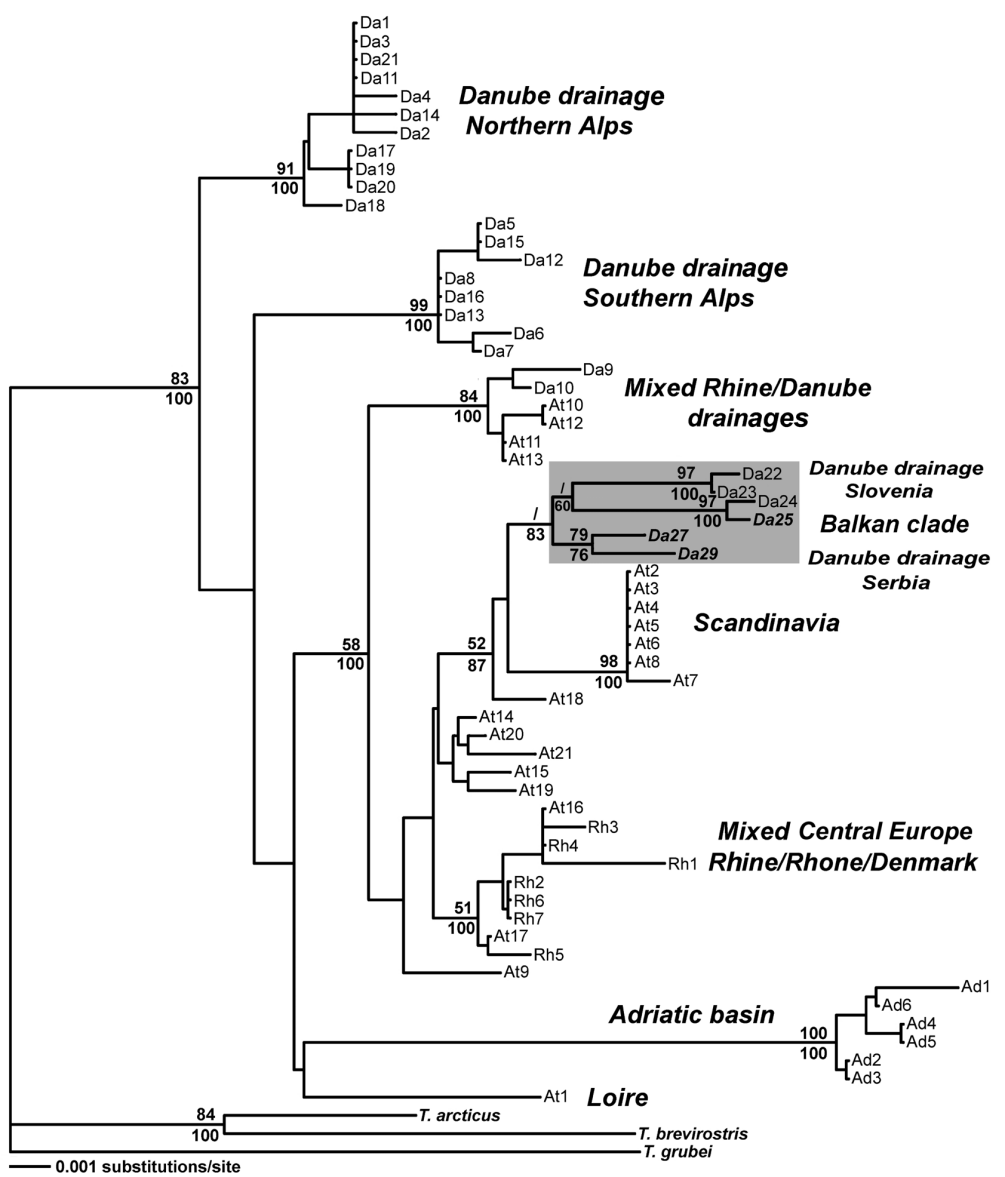
**Phylogeny of mtDNA control region haplotypes**. NJ phylogram based on the data set of Weiss et al. [3] including 58 haplotypes plus three new sequences of the 3'end of the 595 bp fragment of mtDNA CR and 74 bp of flanking tRNA (Phe) from the Serbian part of Danubian drainage; the tree was rooted with *T. grubei, T. brevirostris *and *T. arcticus *sequences of mtDNA CR; node support is shown by per cent bootstraps for NJ (1000 replicates) above, and maximum parsimony consensus (1000) below; italicized taxa represent newly sampled haplotypes

Reconstructing phylogenies by both Neighbour-Joining and parsimony methods revealed that haplotype Da25 and the Slovenian haplotypes from the Sava River drainage area form a sister clade of the new clade containing haplotypes Da27 and Da29 from the Danube River drainage area of Serbia (Figure 2). Both sister clades form the new Balkan clade.

**Figure 3:**
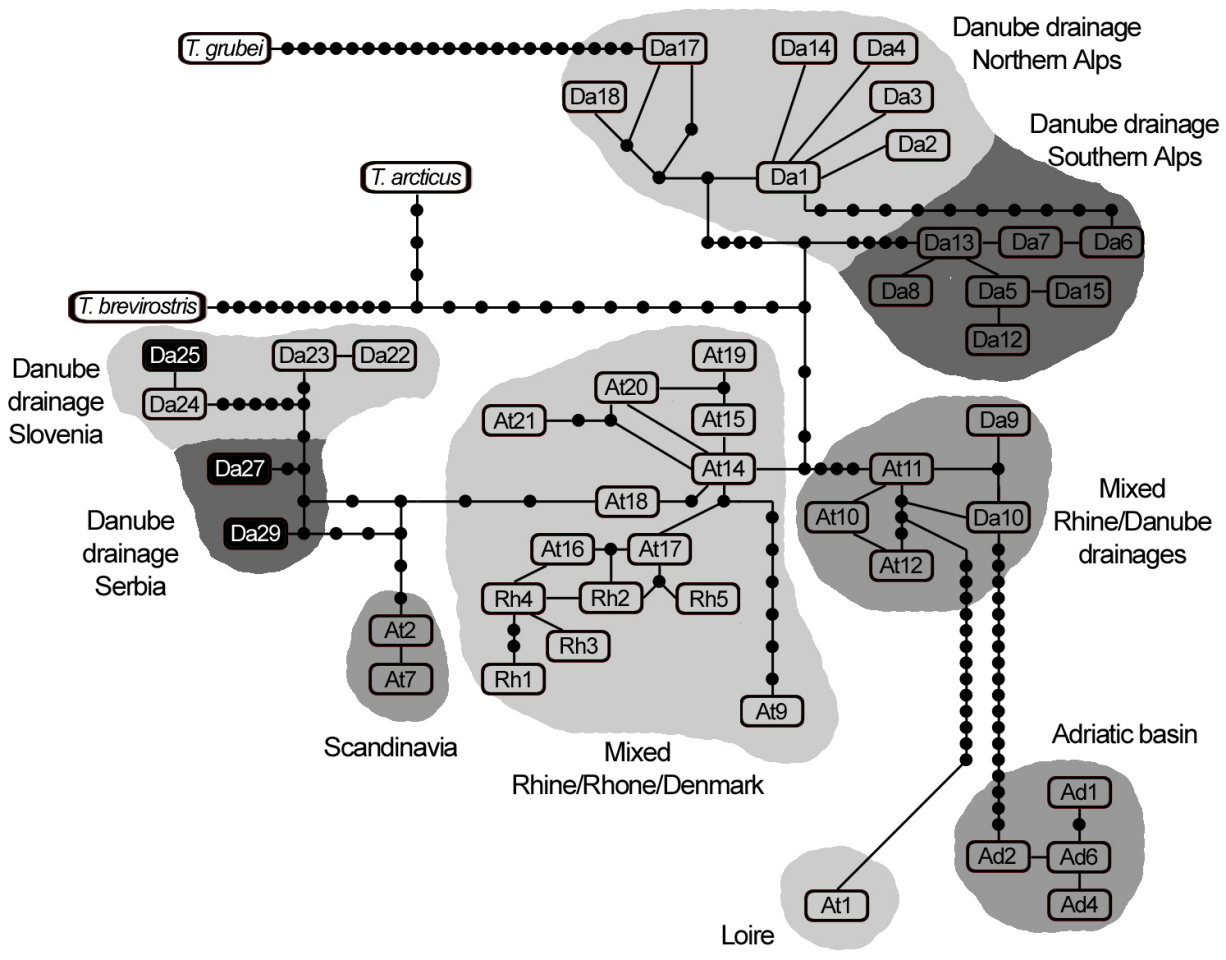
**mtDNA control region haplotype network relating grayling with previously published data **[3]. Lines, regardless of length, represent single mutational events and link the haplotypes; small black circles represent missing or theoretical haplotypes; the three haplotypes found in Serbia are in black

The minimum spanning haplotype network of genealogical relationships among the haplotypes revealed that haplotype Da25 is closest to haplotype Da24 in the Slovenian group of haplotypes in the Sava River drainage area (Figure 3) from which it differed by a single mutation, while it differed by nine or more mutations from other Slovenian haplotypes from the same drainage area (Da22 and Da23). Haplotypes Da27 and Da29 constitute a special group in the network and occupy a position which is most closely related to the Slovenian group of haplotypes from which they differ by six or more mutations.

### Microsatellite marker analysis

Allelic richness ranged from 1.58 to 4.08 and observed heterozygosity ranged from 0.16 to 0.47. The highest levels of allelic richness (4.08), observed (0.47) and expected (0.49) heterozygosities were detected in the Drina River samples, while those of the Rzav and Ibar Rivers had very low levels of heterozygosity. No deviations from Hardy-Weinberg equilibrium were detected (Table 2).

**Table 2:** Genetic diversity indices of microsatellite marker data

Population	Nb	**H**_**E**_	**H**_**n.b.**_	**H**_**O**_	**F**_**IS**_	Ar
1. Rzav	20	0.27	0.27	0.27	0.02	1.75
2. Lim	20	0.29	0.29	0.30	-0.02	2.83
3. Ibar	20	0.15	0.16	0.16	-0.05	1.58
4. Drina	20	0.49	0.50	0.47	0.07	4.08

Nb: number of individuals; H_E_: expected heterozygosity in the population; H_n.b: _non biased heterozygosity; H_O_: observed heterozygosity; F_IS: _values showed no statistically significant deviations from HWE (P < 0.001); Ar: allelic richness

### Bottlenecks

**Figure 4:**
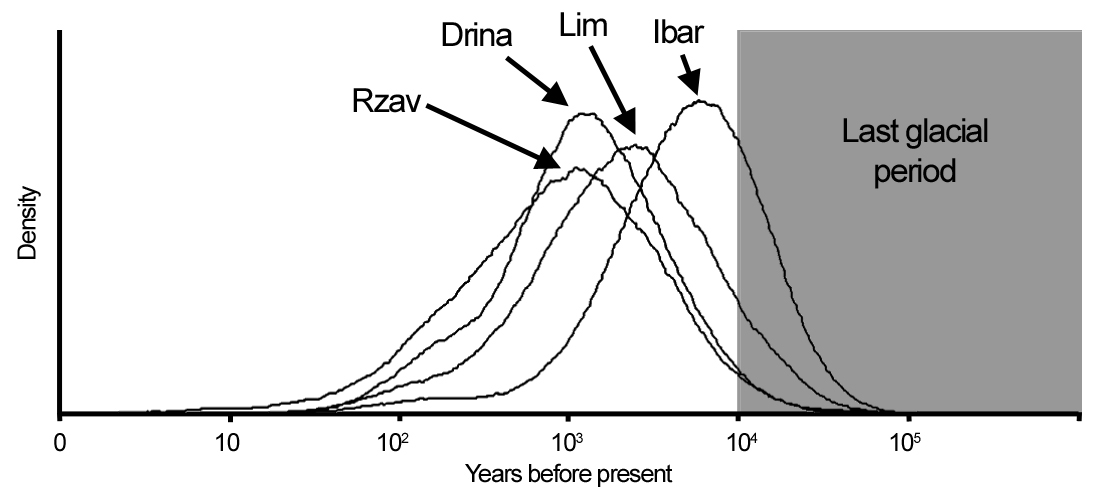
**Estimated time since the start of population decline using MSVAR **[44]** (posterior distribution) based on microsatellite marker data**.

Recent bottleneck was detected in the Rzav River (SMM and TPM), P < 0.05. Mode-shift test also revealed distortion from L-shape allele frequency distribution in the same river. Coalescent analyses assuming an exponential population growth/decline estimated that the population decline started 1000 - 10 000 years ago with the present population sizes representing 0.03-0.44% of the historical sizes (Figure 4).

### Population differentiation, clustering and introgression

**Figure 5:**
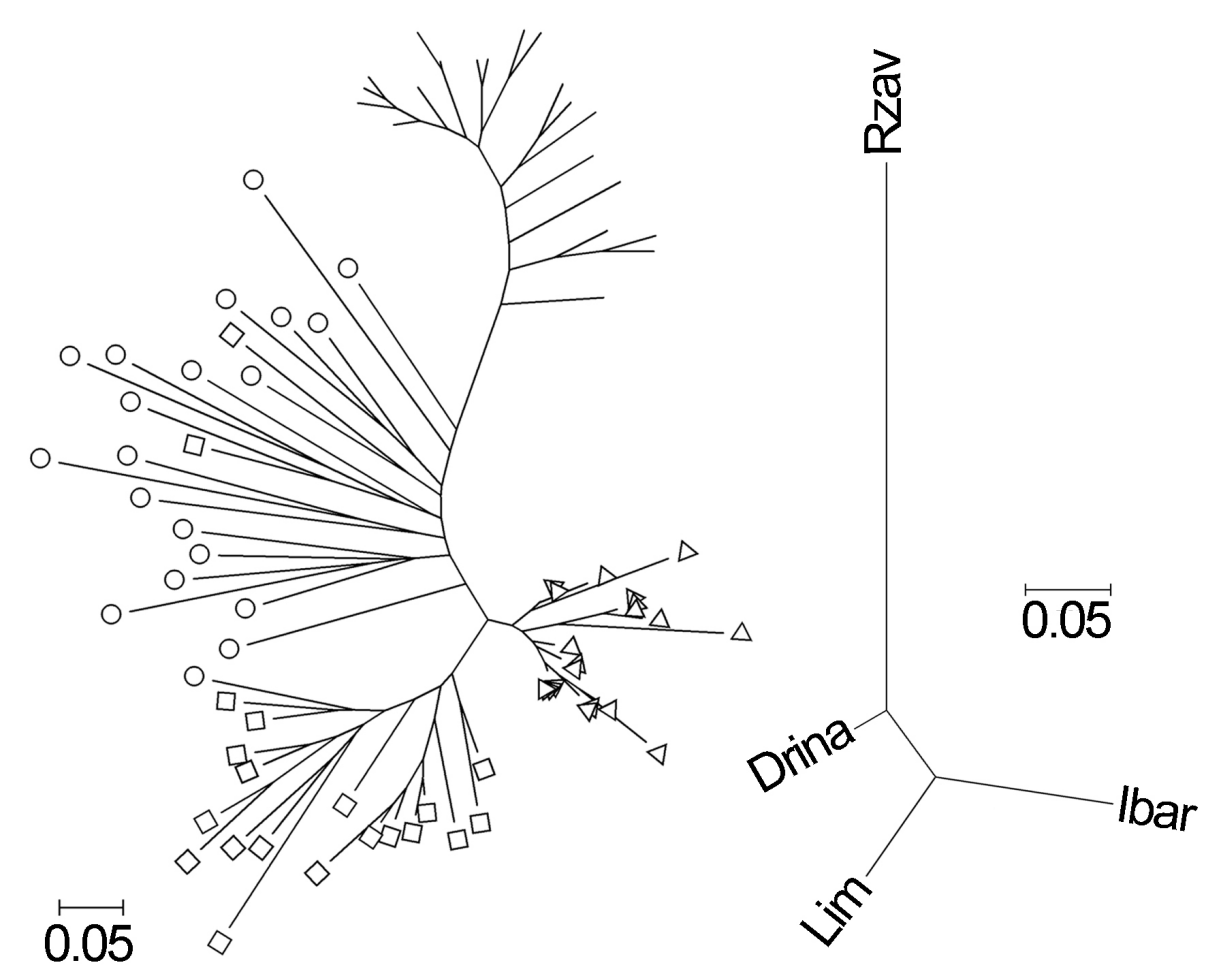
**Neighbour-Joining individuals (left) and population (right) trees based on D_AS _estimated from 12 microsatellite DNA loci**. Individuals from the Drina, Lim and Ibar Rivers are labeled with circles, squares and triangles respectively, Rzav is unlabeled

**Figure 6:**
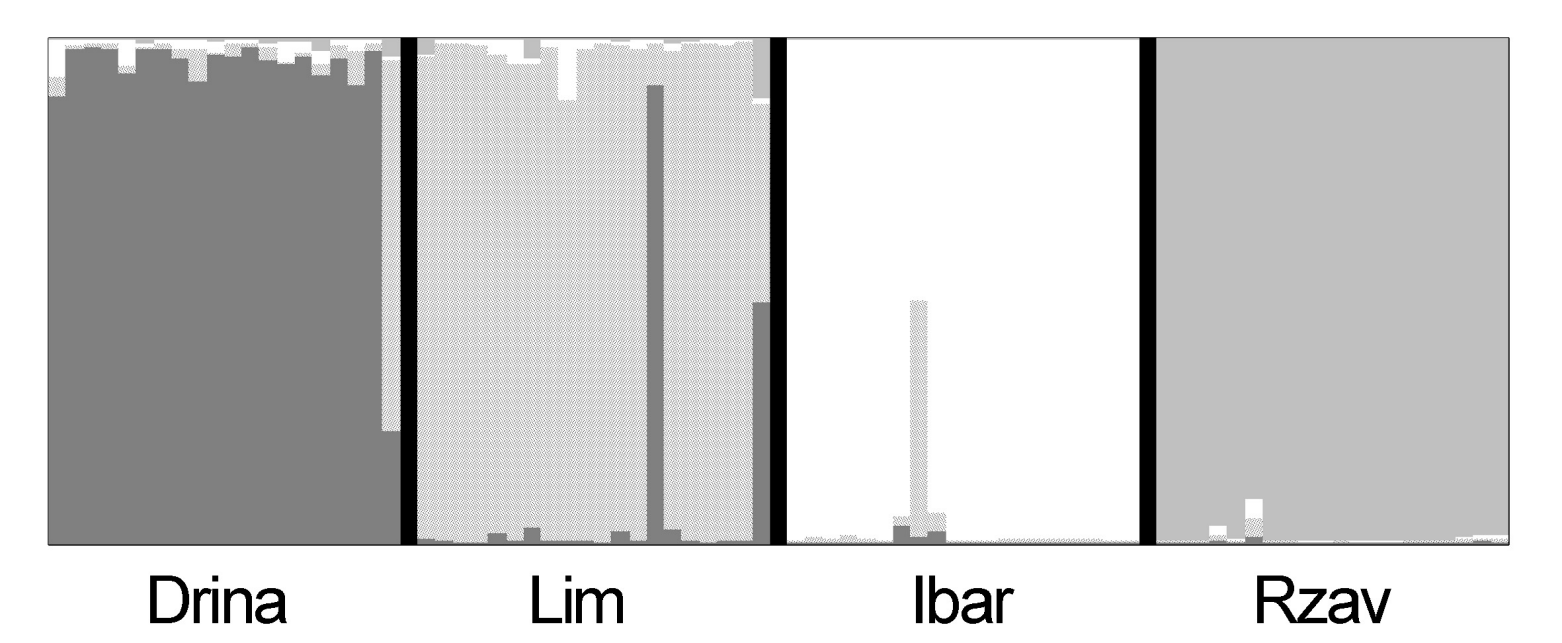
**Estimated population structure as inferred by STRUCTURE analysis of microsatellite marker DNA data**. Black lines separate sampling sites, the most probable K = 4 is based on ΔK method [47]; no further structures were detected in subsequent rounds and within sampling locations (K = 1)

Pairwise F_ST _comparison revealed significant differences among all populations (Table 3). This was also visible in the D_AS _based tree (Figure 5) and in the individual clustering results by Structure, where four clusters corresponded roughly to the four populations from Serbia (Figure 6). Further intra-population partitioning using Structure did not reveal any new cluster and no hybrid population was detected. (See additional file 3: The original data used to perform this analysis).

**Table 3:** Paired values of F_ST _above and D_AS _below the diagonal of microsatellite marker data

	Rzav	Lim	Ibar	Drina
Rzav		0,513*	0,624	0,340*
Lim	0,437		0,358*	0,178*
Ibar	0,480	0,176		0,226*
Drina	0,343	0,146	0,172	

* P < 0.001

## Discussion

### Mitochondrial DNA

Two combined haplotypes, Da27Bal and Da29BoDr are autochthonous for populations from Serbian waters while haplotypes Da25Soc18 and Da25Slo originate from Slovenia (Table 1). The practice of stocking with grayling was common in many European countries [15,25,48]. Despite the absence of written records on stocking in Rzav and Drina Rivers with grayling fry originating from Slovenian hatcheries, testimonies from the older members of the Anglers' Association of Arilje do agree that grayling was introduced into the Rzav River from Slovenia in the 1980's. This is supported by the fact that haplotype Da25 also occurs in Slovenian populations (unpublished data). The 0.75% genetic distance between autochthonous Da27 and Da29 haplotypes for which the mutation rate is estimated at about 1% per million years [3,14,49], suggests that a time period of about 750 thousand years separates the two haplotypes involving probably two independent colonization events. This assumption is supported by the even greater genetic distance (~1.35%) occurring between the two groups of haplotypes (Da22, Da23) and (Da24, Da25) in the Slovenian grayling sub-clade.

Within the Balkan clade, the division between northern (Slovenian) and southern (Serbian) populations is obvious. This northern/southern differentiation has also been recorded in another Danubian salmonid species i.e. huchen (*Hucho hucho*) on the basis of microsatellite data [50].

The grayling Balkan and the Scandinavian clades are sister clades (Figure 2), suggesting a common ancestry in the drainage areas of Black and Caspian seas. This is in agreement with the ancestral character of the grayling populations from a Danubian refugium in relation to the northern populations [3,5]. The intermediate position of the Da29 haplotype on the network between northern Balkan and Scandinavian clades supports this statement. Da29 could be considered as the basal haplotype of the Balkan clade, with the genetic distance between Da29 and other haplotypes ranging between 0.75 and 1.65%, which is equivalent to the time scale of 750 000-1.65 million years when grayling of northern, Slovenian and southern, Serbian clusters differentiated. This is similar to the distance (~1.5%) found between the two northern and southern Alpine clades [3].

Including the ATP6 gene to reconstruct phylogenetic relationships in European grayling clades is not very useful at present because most studies are based on CR or other mtDNA sequences. Analyzing the ATP6 gene in grayling populations of Serbia confirms the division, previously observed from CR sequencing results, into two subclades (the northern i.e. Slovenian and the southern i.e.Serbian) in the Balkan clade defined by the synapomorphy at position 34 of the ATP6 gene (see additional file 2).

Results obtained so far suggest that the ATP6 gene will be a useful marker for future investigations on European grayling populations, since it provides interesting information on genetic variability. This could be important for decisions in conservation and management of grayling populations.

### Microsatellite markers

In terms of microsatellite diversity (allelic richness, observed and expected heterozygosities), populations from this study are comparable to other populations [7,51]. F_ST _pairwise comparisons and Structure analyses reveal a strong divergence between Serbian grayling populations, which is not characteristic of Slovenian grayling in the Danubian drainage [11,52]. Recent bottleneck has been shown in population from the Rzav River, which has suffered a recent decline. In addition, the Rzav River habitat is relatively small and was initially stocked with (presumably) a small number of fish from Slovenia.

Serbian locations represent the furthest grayling habitats from the maximal extent of ice sheets [21] and possibly grayling glacial refugium (or closest to it). Nevertheless the historical decline of Serbian populations is comparable with other European populations analyzed by Swatdipong et al. [24] and is dated between 1000 and 10 000 years ago.

The Rzav population shares 19 out of 21 alleles (90%) with Slovenian populations (Sava, Obrh and Unec combined), while the Drina, Lim and Ibar populations share 47, 56 and 53% of alleles with Slovenian populations, respectively. The two alleles in the Rzav population not shared with Slovenian populations are found in other Serbian populations, which means that there is no Rzav specific allele. This is not surprising since the river was not naturally inhabited by grayling. While stocking of the Rzav River is confirmed both by the mtDNA haplotypes (100% Da25) and the 90% alleles shared with Slovenian populations, the situation in the Drina River is different. Although 40% of the Drina River samples had the Da25 haplotype, hybridization with non-native grayling was not detected by nuclear markers. The percentage of shared alleles with the Slovenian populations was lowest in Drina River (47%), most likely because the population had already the highest genetic diversity prior stocking. Influence of stocking in the Drina River was not detected in its tributary i.e. the Lim River, which is in accordance to the generally low migration rates for the species [6,16,53]. While Lim and Ibar Rivers are inhabited by native non-introgressed grayling of lower genetic diversity, the Drina River population is admixed and the most diverse in the region.

## Conclusions

Serbian grayling populations are genetically distinct from Slovenian and other European populations. In order to preserve their overall genetic diversity and integrity, further stocking of non-native fish from other regions or from allochthonous populations in the Rzav River should be stopped. Populations from the Ibar and Lim Rivers (which show no signs of introgression of non-native grayling), as well as the population from the Drina River should be regarded as native and subject to proper management. The population from the Drina River is the most diverse in this study and the only one with the mtDNA haplotype Da29. It probably represents the most valuable genetic resource in the region. However, any future management such as supplementary stocking of hatchery-reared Drina River grayling should take into consideration genetic testing prior formation of brood stock, because introgression has been detected. Since the area studied here represents only a minor part of the Balkan Peninsula, genetic polymorphism of the grayling within the region as a whole may be even higher, because grayling from the countries adjacent to Serbia (i.e., Montenegro and Bosnia-Herzegovina) have not been studied so far.

## Competing interests

The authors declare that they have no competing interests.

## Authors' contributions

SM performed the laboratory work and wrote the manuscript with assistance from AR and PS. AR conducted the data analyses. VN organized the logistic for the fieldwork, participated in the collection of data and helped to draft the manuscript. PS coordinated and supervised the study. All authors read and approved the final manuscript.

## Supplementary Material

Additional file 1 **Variable nucleotide positions for mtDNA CR haplotypes defined in this study (underlined) and haplotypes from other European clades**. Positions are homologous to the *T. thymallus *haplotype Da1 (Accession number. AF522395) and correspond to the control region (430-1024), and the tRNA phenylalanine gene (1025-1098) Asterisks refer to base pair deletions or insertions, dashes represent concordance with the Da1 haplotype Click here for file

Additional file 2 **Variable nucleotide positions for mtDNA ATP6 gen haplotypes defined in this study**. Positions are homologous to the *T. thymallus *haplotype Soc18 (Accession number. AY779004) and correspond to the ATP6 gene (14-643); dashes represent concordance with the Soc18 haplotype Click here for file

Additional file 3 **Individual microsatellite marker data**. Individual microsatellite marker data in each population for Tables 2 and 3 and Figures 4 to 6 Click here for file
